# Mr. Wakley, London, England

**Published:** 1862-08

**Authors:** 


DEATH OF MR. WAKLEY.—One of the June numbers of the *Lancet* chronicles the death of Mr. Wakley, the founder of that able journal. He died of consumption, at the age of 67, after a life of unwearied labor in the cause of medical reform; and it may be said that to him, more than to any one else, does modern medical journalism owe much that distinguishes it. Mr. Wakley did not confine himself to the rectification of medical abuses. He was a prominent member of the House of Commons, and here, besides defending and upholding, on all occasions, the rights of his medical brethren, he was "determined in his exposure of all popular abuses, and still more in his championship of the neglected poor." "In addition," says the *Lancet*, "to other services that Mr. Wakley rendered to the profession, may be mentioned the part he took in establishing a system of clinical teaching in London. Previous to the appearance of that journal, clinical teaching was unknown. He was the first to publish reports of the proceedings of the various medical societies. This course met with strenuous and long-continued opposition, and it required years of resolute perseverance to bring this kind of reporting to anything like perfection. It is now an important element in professional instruction. Mr. Wakley was always the defender and upholder of the rights and privileges of the surgeons of the united services, and of the Poor-law medical officers. He lent his aid effectually to the reform of the laws affecting lunatics. His name is associated with most of those proceedings which had reference to the welfare of the great body of surgeons in general practice."

He had, for some years previous to his death, abandoned his editorial labors, never, however, failing "in generous regard and affection for the" journal, which has, for many years, exercised so powerful an influence in the medical world. The memory of such a man as Mr. Wakley will long be cherished as the most successful medical reformer of the age.—*Boston Med. and Surg. Journal.*

